# Associations between Prenatal Exposure to Polychlorinated Biphenyls and Neonatal Thyroid-Stimulating Hormone Levels in a Mexican-American Population, Salinas Valley, California

**DOI:** 10.1289/ehp.9843

**Published:** 2007-06-29

**Authors:** Jonathan Chevrier, Brenda Eskenazi, Asa Bradman, Laura Fenster, Dana B. Barr

**Affiliations:** 1 Center for Children’s Environmental Health Research, School of Public Health, University of California, Berkeley, California, USA; 2 California Department of Health Services, Division of Environmental and Occupational Disease Control, Richmond, California, USA; 3 National Center for Environmental Health, Centers for Disease Control and Prevention, Atlanta, Georgia, USA

**Keywords:** cytochrome P450, enzyme inducers, *in utero*, microsomal enzymes, neonatal, polychlorinated biphenyls, prenatal, thyroid hormone, TSH, UDP-glucuronosyltransferase

## Abstract

**Background:**

Studies have reported that prenatal exposure to polychlorinated biphenyls (PCBs) may alter neurodevelopment in both humans and animals. Furthermore, prenatal exposure to some PCB congeners and commercial mixtures has been shown to decrease free and total thyroxine (T_4_) blood levels in animals. Because thyroid hormones (TH) are essential for normal neurologic development, it has been suggested that the deleterious neurodevelopmental effect of PCBs may occur through TH disruption. PCBs may in turn affect TH levels by inducing the microsomal enzyme uridinediphosphate glucuronosyltransferase (UDP-GT), which is involved in TH elimination.

**Objectives:**

Our goals were to group PCB congeners based on their potential to induce microsomal enzymes in animals, and to examine the relationship between neonatal TSH levels and prenatal exposure to PCB congeners grouped according to their structure and potential mechanisms of action.

**Methods:**

We measured the concentration of 34 PCB congeners in serum samples collected from 285 pregnant women and the thyroid-stimulating hormone (TSH) levels in their children’s blood collected shortly after birth.

**Results:**

We found no association between the sum of PCB congeners, the toxic equivalents, or structure-based groupings (mono- or di-*ortho* substituted congeners), and TSH blood concentration. However, we found a positive association between the sum of congeners suspected to be UDP-GT inducers (more specifically cytochrome P450 2B inducers) in animals and neonatal TSH levels. In individual congener analyses, PCBs 99, 138, 153, 180, 183, 187, 194, and 199 were positively associated with neonatal TSH levels after adjustment for covariates. PCBs 194 and 199 remained significant after adjustment for multiple hypothesis testing.

**Conclusions:**

Our results support grouping PCB congeners based on their potential mechanism of action of enzyme induction when investigating associations with TH. Findings also suggest that PCBs affect TH homeostasis even at the low background level of exposure found in the CHAMA-COS (Center for the Health Assessment of Mothers and Children of Salinas) population.

Polychlorinated biphenyls (PCBs) are synthetic chemicals that were widely used in electrical transformers, inks, plastics, and other consumer products. A total of 209 PCB congeners can be produced depending on the number of chlorine atoms and their position on the biphenyl structure. PCBs are lipophilic and persistent in the environment and bioaccumulate in animals and humans. Although PCBs have been banned in the United States since the 1970s, they can still be measured in most U.S. residents [Centers for Disease Control and Prevention (CDC) 2005].

Several epidemiologic studies have reported that prenatal exposure to PCBs is associated with poorer neurodevelopment in neonates, toddlers, and school-age children (Grandjean et al. 2001; Jacobson and Jacobson 1996; Jacobson et al. 1985, 1990; Koopman-Esseboom et al. 1996; Rogan and Gladen 1991; Rogan et al. 1986). These findings are consistent with animal studies in rodents and rhesus monkeys, which found that *in utero* exposure to PCBs was related to poorer discrimination reversal learning and spatial learning (Levin et al. 1988; Schantz et al. 1989; Widholm et al. 2004). Together with altered neuronal Ca^2+^ signaling (Wong et al. 1997) and reduced dopamine levels (Seegal et al. 1991), disruption of thyroid hormone (TH) homeostasis, which is essential for normal brain development, has been proposed as a potential mechanism for the deleterious neurodevelopmental effects of PCBs. In animals, hypothyroidism affects neuronal proliferation, migration, myelination, and synaptogenesis (Ibarrola and Rodriguez-Pena 1997; Nicholson and Altman 1972; Rami and Rabie 1990). In humans, maternal thyroid status may be of critical importance for fetal neurodevelopment (Haddow et al. 1999; Man and Serunian 1976; Pop et al. 1999), and iodine deficiency–related hypothyroidism is a known cause of cretinism, the leading preventable cause of mental retardation worldwide (Dunn 1993). Congenital hypothyroidism, associated with a variety of pathologies resulting in insufficient TH levels, can also lead to neurodevelopmental problems (de Vilder and Vulsma 2000; Kempers et al. 2006). For this reason, TH levels are routinely measured as part of neonatal screening programs so that TH supplements are administered promptly if necessary.

Several studies suggest that PCBs may disrupt TH levels. PCBs, and especially their hydoxylated metabolites (OH-PCBs), are structurally similar to thyroxine (T_4_). Some PCB congeners reportedly induce the microsomal enzyme uridinediphosphate glucuronosyltransferase (UDP-GT), which catalyzes the glucuronidation of T_4_ (Hood et al. 2003; Liu et al. 1995). Additionally, dioxin-like PCB congeners can bind to the aryl hydrocarbon receptor, resulting in the induction of the cytochrome P450 CYP1A1 as well as the UDP-GT isoenzyme UGT1A6 (methyl-cholanthrene-like inducers), which together with UGT1A1 is responsible for the glucuronidation of T_4_ in rats (Visser et al. 1993). Other PCB congeners have a phenobarbital-like induction pattern which is characterized by the induction of CYP2B and UGT1A1 (Sugatani et al. 2001). PCBs inducing CYP1A and CYP2B are therefore likely to also induce UDP-GT.

In animals, *in utero* exposure to PCB mixtures as well as to individual PCB congeners decreases free and total T_4_ blood levels. Morse et al. (1996) exposed rats to the commercial PCB mixture Aroclor 1254 from days 10 to 16 of gestation and found a dose-related reduction in circulating total and free T_4_ in fetuses on gestation day 20 and pups on postnatal days 4 and 21. Similar results were obtained with PCB-77 in mice (Darnerud et al. 1996) and OH-PCBs in rats (Meerts et al. 2002).

Human studies suggest a relationship between PCBs and TH, but results differ depending on the PCB congeners studied, the grouping schemes used, and whether TH and PCB levels have been examined in the placenta, maternal, cord, or infant blood. For example, Osius et al. (1999) reported positive associations between PCB-118 and thyroid-stimulating hormone (TSH) both measured in children (*n* = 320) as well as negative associations between PCBs 138, 153, 180, 183, and 187, and the sum of seven congeners, and free triiodothyronine (T_3_). Takser et al. (2005) found negative associations between PCBs 138, 153, 180, and the sum of 11 congeners, and total T_3_ in 101 pregnant women, but did not find associations with TSH. In another study, the sum of PCB congeners measured in 160 maternal serum and breast milk samples was not associated with cord blood total T_4_, free T_4_, or TSH levels (Longnecker et al. 2000). Ribas-Fito et al. (2003) measured seven PCB congeners (28, 52, 101, 118, 138, 153, and 180) in 70 cord blood samples and found no significant association of their individual or summed concentrations with TSH in 3-day-olds. In the only study to measure TSH in neonates in relation to maternal PCB exposure, Koopman-Esseboom et al. (1994) reported significant positive correlations between the nonplanar PCB toxic equivalent (TEQ) measured in breast milk and TSH levels in 2-week-olds and between the planar PCB TEQ, the dioxin TEQ, and the PCB–dioxin TEQ, and infant TSH levels at 2 weeks and 3 months of age.

Animal studies suggest that PCB congeners differ in their mechanisms of action and in their toxicologic potencies (Desaulniers et al. 1997; Khan and Hansen 2003; Li et al. 2001) leading some researchers to develop mechanism-based congener groupings. One such grouping is based on congeners’ dioxin-like properties using a method to weigh them by their toxic equivalencies relative to 2,3,7,8-tetrachlorodibenzo-*p*-dioxin (TCDD) (Van den Berg et al. 2006). Wolff et al. (1997) proposed three groups of PCB congeners based on potential mechanisms of action, structure–activity considerations as well as occurrence in house dust and human samples: Group 1 included PCB congeners that are potentially estrogenic; group 2 included congeners that are potentially antiestrogenic, immunotoxic, and dioxin-like; and group 3 included microsomal enzyme inducers. To date, PCB congeners have not been grouped based on their potential to disrupt TH homeostasis.

Although other mechanisms may be involved, current evidence seems to support the hypothesis that the reduced T_4_ levels caused by PCBs in animals occurs at least in part through the induction of UDP-GT. An increase in biliary T_4_–glucuronide excretion has been reported following treatment of rodents with PCBs (Bastomsky 1974). Furthermore, administration of Aroclor 1254 to thyrodectomized rats implanted with osmotic pumps delivering T_4_ resulted in a reduction of total T_4_ and free T_4_ by 75% and 70%, respectively, supporting an extrathyroidal mechanism of action (Barter and Klaassen 1992). Among all UDP-GT inducers tested in the study, UDP-GT activity was negatively correlated with serum total and free T_4_ levels, supporting the hypothesis that induction of this microsomal enzyme contributes to the PCB-related reduction in circulating TH levels.

The purpose of the present investigation is twofold: to group PCB congeners based on their potential to induce microsomal enzymes in animals; and to examine the relation between neonatal TSH and prenatal exposure to PCB congeners grouped according to their structure and potential mechanisms of actions.

## Methods

### Participants

Data for this study were collected as part of the Center for the Health Assessment of Mothers and Children of Salinas (CHAMACOS), a longitudinal birth cohort study investigating environmental exposures and the health of pregnant women and children residing in the agricultural Salinas Valley, California. Pregnant women planning to deliver at Natividad Medical Center (NMC), a county hospital located in Salinas, California, and receiving prenatal care in this hospital or at one of five clinics of Clinica de Salud del Valle de Salinas were screened for enrollment between October 1999 and October 2000. Women were eligible to participate if they were ≥18 years of age, had completed < 20 weeks gestation, spoke English or Spanish, and were MediCal eligible. A total of 601 women agreed to participate in the study, resulting in 538 live births. Excluded from these analyses were participants with insufficient serum volume for PCB analyses (*n* = 118), no locatable TSH data or only diagnostic information (*n* = 111), missing neonatal age at the time of blood draw (*n* = 22), and twins (*n* = 2). Women included in analyses (*n* = 285) were similar to those excluded except in that they had spent less time in the United States than excluded women. This study was approved by the University of California, Berkeley, Committee for the Protection of Human Subjects. All participants gave written informed consent before inclusion in the study.

### Interviews

Participants were interviewed during pregnancy as well as shortly after delivery in English or Spanish by bilingual, bicultural staff. Sociodemographic information collected included maternal age, family income, the number of people supported by this income, country of birth, and number of years lived in the United States. Information on alcohol, tobacco, drug, and caffeine consumption as well as agricultural work was also collected.

### Measurement of neonatal TSH

TSH is routinely measured by the California Department of Health Services Genetic Diseases Branch as part of the state’s Neonatal Screening Program. Samples were collected by heel stick and deposited on a filter paper, which was left to dry at room temperature. Dried blood spots were then analyzed with a solid-phase, time-resolved sandwich fluoroimmunoassay (AutoDELFIA; PerkinElmer, Wellesley, MA) using a lanthanide metal europium (Eu) label. These data were abstracted from medical records. On average, blood spot samples were collected 25 hr after birth (range, 4–121 hr).

### Measurement of PCBs in maternal serum

Blood samples were collected by venipuncture at the end of the second trimester (mean ± SD gestational age = 26.1 ± 2.9 weeks) and shortly before delivery. Delivery samples were only included in the few cases when the second-trimester sample was not collected (*n* = 19). Samples were processed at NMC and stored at −80°C until shipment on dry ice to the CDC in Atlanta, Georgia, for analysis. We measured a total of 34 PCB congeners (International Union for Pure and Applied Chemistry nos. 18, 28, 44, 49, 52, 66, 74, 87, 99, 101, 118, 128, 138, 146, 149, 151, 153, 156, 157, 167, 170, 172, 177, 178, 180, 183, 187, 189, 194, 195, 196, 199, 206, 209) by high-resolution gas chromatography/high-resolution mass spectrometry with isotope dilution quantification based on methods previously published (Barr et al. 2003). Quality control samples were included in each run. Simple substitution methods, which consist of assigning values when measurements are below the limit of detection (LOD) such as LOD/2 or LOD/√2, may bias results when detection frequencies are < 90–95% (Lubin et al. 2004). Values below the LOD were therefore randomly imputed from a log-normal distribution whose parameters were determined by maximum likelihood estimation. This method generally produces unbiased parameter estimates (Lubin et al. 2004). LODs for PCBs ranged between 0.01 and 1.92 ng/g lipids. Statistical analyses were restricted to PCB congeners with a detection frequency > 75% and included PCBs 18, 28, 44, 49, 52, 66, 74, 99, 101, 118, 138, 146, 153, 156, 180, 183, 187, 194, and 199.

Triglycerides and total cholesterol were determined using standard enzymatic methods (Roche Chemicals, Indianapolis, IN). Total blood lipid concentrations were then calculated using the method reported by Phillips et al. (1989). PCB values were lipid-adjusted for all analyses by dividing serum PCB concentrations by total blood lipid concentrations.

### PCB groupings

PCB congeners were grouped according to previously proposed structure-based and mechanism-based groupings (Van den Berg et al. 2006; Wolff et al. 1997). Structure-based groupings were generated by summing the individual levels of mono-*ortho* (PCBs 28, 66, 74, 118, 156, 157, 167, and 189) and di-*ortho* substituted (PCBs 18, 44, 49, 52, 87, 99, 101, 128, 138, 146, 153, 172, 180, and 194) PCBs. The mechanism-based methods included the three groupings proposed by Wolff et al. (1997) and the TEQ method. We calculated TEQs using the World Health Organization’s toxic equivalency factors (TEFs) (Van den Berg et al. 2006). In addition, we created *a priori* another mechanism-based grouping of PCBs according to their ability to induce UPD-GT, CYP1A [or 7-ethoxyresorufin-*O*-deethylase (EROD)] and CYP2B [or 7-pentoxyresorufin-*O*-dealkylase (PROD)] in mammals. PCB congeners likely to induce these enzymes were identified through a search in PubMed (http://www.ncbi.nlm.nih.gov/sites/entrez) and through referenced articles (Table 1). PCB congeners were considered enzyme inducers if a continuous linear or nonlinear dose response could be identified without otherwise overt toxic effects. Doses necessary to induce microsomal enzymes vary among animal species and strains tested (Boobis et al. 1990). We considered a dose–response relationship in at least one strain for UDP-GT, CYP1A, or CYP2B as an indication of possible UDP-GT induction in humans. PCB congeners in the final grouping included PCBs 52, 99, 101, 118 153, 156, 157, 167, 180, 183, 187, 189, 194, and 199. Because dioxin-like PCBs induce CYP1A, the enzyme inducers grouping included these congeners.

### Statistical analyses

We used Pearson’s correlations to evaluate the interrelationship of PCB congeners. We then used one-way analysis of variance (ANOVA) to compare PCB levels by demographic characteristics. We constructed multiple linear regression models to test the association of both individual PCB congeners as well as PCB groupings with neonatal TSH levels. Covariates considered in regression models included (categorized as shown in Table 2 or as indicated below): maternal age (continuously), race, country of birth, marital status (married or not), years of education, prepregnancy body mass index (BMI; continuously); cigarette, alcohol, and caffeine consumption during pregnancy (none vs. any); gestational age at time of blood collection for PCB analysis (continuously); and neonate’s birth weight (continuously), sex, gestational age at birth (continuously), birth order (continuously), and age (in hours) at the time of heel stick (continuously). Covariates were selected for final models if they were related with the outcome (*p* < 0.20); final models included neonatal age at the time of heel stick, gestational age at birth, infant birth weight, sex, and mother’s prepregnancy BMI.

We also considered the potential confounding effect of other environmental chemicals such as lead, organophosphate pesticides, and other organochlorine compounds including *o,p*′-dichlorodiphenyltrichloroethane (DDT), *p,p*′-DDT, *p,p*′-dichlorodiphenyl-trichloroethylene (DDE), β- and γ-hexachloro-cyclohexane, dieldrin, hexachlorobenzene, heptachlor epoxide, mirex, oxychlordane, and *trans*-nonachlor. Organochlorine compounds were measured concurrently with PCBs using the method described above and were lipid-adjusted. We determined exposure to organophosphate pesticides by averaging the concentration of dialkyl phosphate metabolites measured in urine by high-resolution gas chromatography–tandem mass spectrometry with isotope dilution quantification; these samples were collected twice during pregnancy (mean gestational age, 13 and 26 weeks) (Bradman et al. 2005; Bravo et al. 2004; Eskenazi et al. 2004). We measured lead in maternal and cord blood using graphite furnace atomic absorption spectrophotometry.

TSH levels surge at birth and decrease sharply within the first days of life, the period during which blood was collected for TSH analysis. Neonate’s age at the time of blood collection (expressed in hours) was negatively associated with both PCBs and TSH in our data. Log_10_(age) maximized the correlation between age at the time of blood collection and log_10_(TSH) and was entered as such in the models (*r* = −0.6, *p* < 0.001). As an alternative, we also age-standardized TSH levels based on data obtained from the neonatal screening program administered by the Genetics Disease Branch of the California Department of Health (http://www.dhs.ca.gov/pcfh/gdb/html/NBS/) (*n* = 1,330,213).

The concentrations of the different PCB congeners are usually highly intercorrelated, so the association of a specific grouping with neonatal TSH may be attributed to uncontrolled confounding by PCB congeners not included in models. Therefore, we also included the sum of those PCB congeners that were not part of any particular grouping in each model.

As described above, we ran multiple models to examine associations of neonatal TSH with individual PCB congeners. The Bonferroni adjustment method may be too conservative when a large number of tests are carried out. We therefore used the bootstrap-based single-step maxT multiple testing procedure proposed by Dudoit et al. (2004), which adjusts for multiple hypothesis testing while accounting for the correlation between exposures by estimating the joint distribution of test statistics (multtest package in R; R Foundation for Statistical Computing, Vienna, Austria).

Adding congeners assumes an equal potency for every component of a given grouping, which may not be appropriate. Thus, we also analyzed the data by using principal-component analysis to summarize both the group of enzyme inducers and congeners that have not been identified as enzyme inducers. Scores for each group’s first component (defined as the loadings-weighted sum of PCB congeners) were then entered as the independent variable in a multiple linear regression model along with the covariates identified above.

Missing values for PCB congeners were imputed based on a nearest neighbor (Euclidian distance) method (impute package in R). Values for environmental exposures (including PCBs) and TSH were expressed on the log_10_ scale for statistical analyses. Analyses were performed with Intercooled STATA, version 8.1 (StataCorp., College Station, TX) and R, version 2.3.0 (R Foundation for Statistical Computing, Vienna, Austria).

## Results

Most participating women were young, Latina, born in Mexico, and had little education (Table 2). Almost all spoke Spanish at home (94%), and most were below the federal poverty line (61%) and had at least one household member working in agriculture (76%). There were slightly more male than female children (52 vs. 48%). Approximately 3% of the newborns had a low birth weight (< 2,500 g), and 8% were premature (< 37 weeks gestation).

TSH levels were within the reference range for all children (≤25 mIU/L) with a geometric mean of 5.7 mIU/L [95% confidence interval (CI), 5.3 to 6.1]. Gestational age at birth and birth weight were positively associated with TSH levels (data not shown). No other covariates were related to TSH levels.

The sum of those PCB congeners with a detection frequency > 75% increased with maternal age (*p* < 0.001) and with the number of years of education (*p* < 0.05; Table 3). Levels were also higher in women who smoked during pregnancy, but the difference was not statistically significant.

Only PCBs 183 (β= 0.11; 95% CI, 0.02 to 0.20) and 199 (β= 0.10; 95% CI, 0.01 to 0.20) were significantly positively related to neonatal TSH levels in univariate regressions (data not shown); however, as shown in Table 4, 6 of the 19 PCB congeners detected in > 75% of the samples—namely PCBs 101, 180, 183, 187, 194, and 199—were found to be significantly related to TSH levels, after adjustment for neonatal age at the time of blood draw (β= 0.08–0.14, *p* < 0.05). Three additional congeners, PCBs 99, 138, and 153, were significantly positively related to TSH after adjustment for all covariates (PCB-99: β = 0.11; 95% CI, 0.02 to 0.21; PCB-138: β = 0.09; 95% CI, 0.01 to 0.18 and PCB-153: β = 0.08; 95% CI, 0.00 to 0.17).

PCBs 194 and 199 remained significantly associated with TSH levels after adjustment for multiple hypothesis testing (PCB-194: β = 0.12; 95% CI, 0.01 to 0.24; PCB-199: β = 0.14; 95% CI, 0.02 to 0.25), whereas PCBs 101, 183, and 187 almost reached statistical significance (PCB-101: β = 0.09; 95% CI, −0.01 to 0.18; PCB-183: β = 0.13; 95% CI, −0.01 to 0.23; PCB-187: β = 0.09; 95% CI, −0.01 to 0.20).

**Figure 1:**
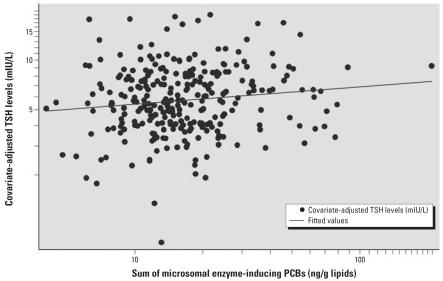
Association between covariate-adjusted TSH levels and the sum of microsomal enzyme-inducing PCB congeners in neonates (*n* = 285).

Total PCB levels, structure-based groupings (mono-*ortho* and di-*ortho* substituted PCBs), and the TEQ of dioxin-like PCBs were not significantly associated with neonatal TSH levels (Table 4). However, the sum of PCBs specifically hypothesized to induce T_4_-metabolizing enzymes was positively associated with neonatal TSH levels; each 10-fold increase in the sum of enzyme-inducing PCBs was associated with a 29% (95% CI, 2 to 62%) increase in TSH (computed from Table 4). The association was linear on a log–log scale, as shown in Figure 1. Including the sum of PCBs not found to be enzyme inducers in the same model or removing extreme values did not materially alter results (data not shown). The principal-component analysis–computed factor summarizing the serum level of potential enzyme inducers (accounting for 64% of the variance) was also significantly associated with neonatal TSH (β= 0.015, 95% CI, 0.005 to 0.024), whereas the factor representing noninducers (accounting for 63% of the variance) was not (β= 0.006, 95% CI, −0.005 to 0.017). Furthermore, the sum of congeners included in group 3 of the classification proposed by Wolff et al. (1997), which is also based on enzyme induction, was significantly associated with TSH levels (β= 0.11; 95% CI, 0.02 to 0.20). The fact that we found a significant association with the sum of all potential enzyme inducer PCBs but not with the TEQ suggests that CYP2B inducers were primarily associated with TSH. This was confirmed when we grouped PCBs by specific enzyme induced (CYP2B/PROD inducers: β = 0.11; 95% CI, 0.01 to 0.21).

Results above were similar whether neonate’s age at the time of TSH measurement was controlled for by including the variable as a covariate in models or by standardizing with the use of an external population (California Department of Health Services, Genetic Diseases Branch). The method used to account for blood lipids (including it as a covariate or expressing PCBs on a lipid basis) did not appreciably alter results.

## Discussion

Results from this study suggest that prenatal exposure to PCB congeners that induce CYP2B in animals is positively associated with TSH levels in children shortly after birth. In animals, UDP-GT (UGT1A1) is induced concurrently with CYP2B following exposure to phenobarbital-like compounds, which may explain the observed association (Sugatani et al. 2001). However, we found no association between levels of TSH and total PCBs, PCBs grouped by structure (mono-and di-*ortho*–substituted PCBs), or dioxin-like PCBs (TEQ). These findings were observed in the CHAMACOS population, which had low exposure to PCBs compared with the general U.S. population (CDC 2005). The median PCB-153 concentration, for instance, was 5.6 times lower in our participants than in the NHANES (National Health and Nutritional Examination Survey) sample (5.4 vs. 30.1 ng/g lipids).

Our findings differ from those of Koopman-Esseboom et al. (1994), who found associations between maternal dioxin-like PCBs (TEQ) and neonatal TSH levels, and of Wang et al. (2005), who reported associations between the placental dioxin/PCB TEQ and cord blood TSH levels. Although we may have underestimated the TEQ in our population because we did not measure the levels of two key dioxin-like PCBs (PCBs 126 and 169), the exposure level in the Dutch study appeared to be substantially higher (about 20 times) than in the current study (Longnecker et al. 2003). Our results also differ from those of previous studies that found no association of TSH levels with individual PCB congeners (Ribas-Fito et al. 2003; Takser et al. 2005). However, our findings are consistent with those of Longnecker et al. (2000), Takser et al. (2005), and Ribas-Fito et al. (2003), who did not find an association between the sum of all PCB congeners measured and TSH levels in pregnant women, neonates, and cord blood. Our results also agree with the lack of association between the structure-based grouping of mono-*ortho*–substituted PCB congeners and TSH levels, as reported in two previous studies (Takser et al. 2005; Wang et al. 2005).

Serum concentrations of PCB congeners are highly intercorrelated, yet individual congeners may differ in their health effects and mechanisms of action. Except for a study by Wolff and Toniolo (1995), who investigated associations with breast cancer, we are not aware of any other study that grouped PCBs based on the mechanism of enzyme induction. To date, no study examining the potential of PCBs to disrupt TH has grouped congeners based on their potential to specifically disrupt TH.

One of the main strengths of this study is that we attempted to characterize *a priori* PCB congeners based on their potential to affect TH. We grouped PCBs based on evidence from animal studies suggesting the potential of specific PCB congeners to induce UDP-GT. We also considered the potential confounding effect of a large number of demographic and environmental covariates. Our findings were further supported by principal-component analysis, with the first factor summarizing enzyme-inducing congeners being significantly associated with neonatal TSH levels whereas the factor summarizing other congeners was not. Strengths also include the control for multiple hypothesis testing and our use of distribution-based imputation techniques for values below the LOD.

There are limitations to the method we propose, some of which stem from limited data. First, we could not find published data for seven of the 19 congeners commonly found in the CHAMACOS population (detection frequency > 75%). Also, our method does not consider other potential mechanisms of action by which PCBs could affect TH levels, including the binding of PCBs to transthyretin and displacement of T_4_ (Chauhan et al. 2000), increased liver T_4_ uptake and decreased pituitary sensitivity to thyroid-releasing hormone (Khan and Hansen 2003), altered T_4_ and T_3_ synthesis (Collins and Capen 1980), and inhibited thyroid gland response to TSH (Byrne et al. 1987). Summing the concentrations of PCB congeners also assumes equal potencies, which may not be appropriate. Summarizing the grouping with principal-component analysis–derived factors, though avoiding the equal potencies assumption of the summation method, still assumes no synergistic effect. An ideal grouping would consider all potential mechanisms of action, different relative potencies, and interactions. One strategy to improve our method might be to develop a TEQ-like weighing system (Van den Berg et al. 2006). Such a scheme would require more congener-specific data and would need to consider whether a simple linear additive model appropriately represents observed effects at environmentally relevant doses.

In summary, we report a positive association between neonatal TSH levels and prenatal exposure to PCBs reported to induce microsomal enzymes (specifically CYP2B) and suspected to induce UDP-GT in animals but not with the sum of all PCB congeners, or PCBs grouped according to their dioxin-like activity or structure. This is the largest study to date investigating prenatal exposure to PCBs and neonatal TSH. If replicated, our findings would support the hypothesis that not all PCB congeners disrupt thyroid hormones, and would argue against summing all PCB congeners. However, they would support grouping PCB congeners based on their potential mechanism of action of UDP-GT induction. Our results also suggest that PCBs affect TH homeostasis even at the low background level of exposure found in the CHAMACOS population. Although TSH remained within the reference range, previous animal and human studies suggest that maternal hypothyroxinemia (low free T_4_ and normal TSH levels) during early pregnancy adversely affects neurodevelopment (Morreale de Escobar et al. 2000; Pop et al. 1999). Future studies should examine whether TH levels within the reference range in neonates may be related to neurodevelopment.

## Figures and Tables

**Table 1:** References supporting microsomal enzyme induction by individual PCB congeners in animals.

	Microsomal enzymes		
PCB congener	UDP-GT	CYP1A/EROD	CYP2B/PROD
42			Connor et al. 1995
47			Connor et al. 1995
52			Connor et al. 1995
77	Desaulniers et al. 1997; Seo et al. 1995; Visser et al. 1993	Chu et al. 1995; Morse et al. 1995; Seo et al. 1995	
99			Connor et al. 1995
101			Connor et al. 1995
105	Chu et al. 1998	Chu et al. 1998	Chu et al. 1998; Connor et al. 1995
118		Chu et al. 1995; Connor et al. 1995; Kuriyama et al. 2003	Connor et al. 1995
126	Craft et al. 2002; Seo et al. 1995; Van Birgelen et al. 1995	Chu et al. 1994; Craft et al. 2002; Desaulniers et al. 1999; Seo et al. 1995; Van Birgelen et al. 1995	
128		Lecavalier et al. 1997	
149			Li et al. 2001
153	Craft et al. 2002	Chu et al. 1996; Desaulniers et al. 1999	Connor et al. 1995; Craft et al. 2002; Desaulniers et al. 1999; Ikegwuonu et al. 1996
154			Connor et al. 1995
156	Van Birgelen et al. 1995	Van Birgelen et al. 1994	Van Birgelen et al. 1994
163			Connor et al. 1995
169	Morse et al. 1993	Morse et al. 1993	Morse et al. 1993
170			Connor et al. 1995
180			Connor et al. 1995
183			Connor et al. 1995
187			Connor et al. 1995
189			Connor et al. 1995
194			Connor et al. 1995
199			Connor et al. 1995

**Table 2:** Demographic characteristics in a population of pregnant women and their children in the Salinas Valley, CA (*n* = 285).

		Sum PCBs (ng/g lipids)^a^	
	No. (%)	Geometric mean	95% CI
Maternal			
Age (years)			
18–24	142 (50)	54.1	49.2–59.5*#
25–29	89 (31)	59.9	53.1–67.5
30–34	35 (12)	72.9	60.2–88.3
35–45	19 (7)	87.5	72.3–105.9
Race			
White	3 (1)	38.9	9.7–156.3
Latina	275 (96)	59.9	55.9–64.2
Other	7 (2)	67.3	50.2–90.3
Education			
≤6th grade	118 (41)	55.7	50.1–62.0#
7–12th grade	100 (35)	59.4	53.0–66.5
≥High school	67 (23)	68.5	60.1–78.1
Income (% poverty)			
< 100	162 (61)	59.3	54.3–64.7
100–200	96 (36)	60.5	53.7–68.2
> 200	9 (3)	49.1	29.9–80.4
Country of birth			
United States	34 (12)	61.9	50.0–76.5
Mexico	244 (86)	59.8	55.6–64.3
Other	7 (3)	52.0	34.5–78.6
Time in the USA (years)			
≤5	156 (55)	59.0	53.7–64.7
6–10	69 (24)	61.3	53.4–70.4
≥11	60 (21)	60.4	52.5–69.5
Parity			
0	103 (36)	61.6	54.8–69.2
≥1	183 (64)	58.8	54.2–63.9
Smoking during pregnancy			
Yes	12 (4)	68.7	48.5–97.3
No	274 (96)	59.5	55.5–63.7
Infant			
Sex			
Male	147 (52)	58.2	52.9–63.9
Female	138 (48)	61.6	56.0–67.8
Birth weight (g)			
< 2,500	9 (3)	66.8	49.9–89.4
2,500–3,500	150 (52)	58.6	53.3–64.4
> 3,500	126 (44)	60.8	55.0–67.2
Gestational age at birth (weeks)			
< 37	22 (8)	61.4	49.7–75.7
37–42	264 (92)	59.7	55.6–64.1
> 42	0 (0)	—	—

a PCBs with a detection frequency ≥75% were summed.

* *p* < 0.05 ANOVA.

# *p* < 0.05 linear trend by Pearson’s correlation.

**Table 3:** PCB levels (geometric means), detection frequencies, ranges, and LOD ranges in a population of pregnant women in the Salinas Valley, CA.

	No.	LOD range	Detection frequency (%)	Geometric mean	95% CI	Range
∑PCBs^a^ (ng/g)	285	0.02–1.92	100	59.8	56.0–64.0	15.3–323.7
TEQ (pg/g)	285	0.004–0.086	100	0.86	0.75–0.97	< LOD–5.17
Inducers^b^ (ng/g)	285	0.03–1.44	100	38.9	36.3–41.6	10.0–250.3
Mono-*ortho*^c^ (ng/g)	285	0.03–1.11	100	29.4	27.2–31.7	6.4–127.7
Di-*ortho*^d^ (ng/g)	285	0.03–1.44	100	27.6	25.9–29.5	7.3–213.1
Wolff method						
Group 1^e^ (ng/g)	285	0.03–1.92	100	8.3	7.6–9.1	0.4–62.9
Group 2^f^ (ng/g)	285	0.03–1.11	100	13.2	12.3–14.1	2.1–69.1
Group 3^g^ (ng/g)	285	0.04–1.09	100	8.4	7.7–9.1	0.6–138.0
Individual congeners						
PCB-18 (ng/g)	279	0.03–0.88	100	6.5	5.9–7.1	0.9–32.1
PCB-28 (ng/g)	285	0.03–0.79	100	17.4	15.8–19.1	3.1–91.7
PCB-44 (ng/g)	238	0.06–1.92	98.7	2.3	2.1–2.6	< LOD–11.4
PCB-49 (ng/g)	250	0.05–1.40	99.2	1.5	1.4–1.7	< LOD–7.9
PCB-52 (ng/g)	261	0.05–1.89	99.2	3.0	2.7–3.3	< LOD–12.4
PCB-66 (ng/g)	276	0.39–1.02	100	2.8	2.5–3.0	0.4–16.0
PCB-74 (ng/g)	276	0.04–1.11	100	4.1	3.8–4.4	0.7–20.8
PCB-99 (ng/g)	263	0.06–1.09	100	1.8	1.7–1.9	0.5–11.6
PCB-101 (ng/g)	241	0.06–1.44	94.6	0.9	0.8–1.1	< LOD–5.8
PCB-118 (ng/g)	270	0.05–0.86	99.6	3.4	3.2–3.7	< LOD–4.7
PCB-138 (ng/g)	263	0.03–0.65	100	2.5	2.3–2.7	0.2–30.9
PCB-146 (ng/g)	249	0.05–0.60	87.6	0.5	0.4–0.5	< LOD–14.7
PCB-153 (ng/g)	273	0.04–0.70	100	5.6	5.2–6.0	0.3–95.6
PCB-156 (ng/g)	270	0.07–0.55	85.2	0.4	0.4–0.5	< LOD–6.3
PCB-180 (ng/g)	231	0.06–0.96	100	1.5	1.4–1.7	0.3–30.0
PCB-183 (ng/g)	259	0.06–0.47	78.0	0.3	0.3–0.4	< LOD–8.2
PCB-187 (ng/g)	224	0.04–0.71	96.9	0.9	0.8–1.0	< LOD–38.3
PCB-194 (ng/g)	263	0.03–0.50	95.4	0.5	0.5–0.6	< LOD–8.6
PCB-199 (ng/g)	271	0.03–0.62	85.6	0.4	0.3–0.4	< LOD–7.5

a Sum of all PCBs with a detection frequency ≥ 75% (listed above).

b Enzyme inducers include PCBs 52, 99, 101, 118, 153, 156, 180, 183, 187, 194, and 199.

c Mono-*ortho* PCBs include PCBs 28, 66, 74, 118, and 156.

d Di-*ortho* PCBs include PCBs 18, 44, 49, 52, 99, 101, 138, 146, 153, 180, and 194.

e Group 1 includes PCBs 44, 49, 52, 101, 187, and 199.

f Group 2 includes PCBs 66, 74, 118, 138, and 156.

g Group 3 includes PCBs 99, 153, 180, and 183.

**Table 4:** Age-adjusted and fully adjusted associations between prenatal exposure to PCBs and neonatal TSH levels.

	Age-adjusted		Fully adjusted a	
	β	95% CI	β	95% CI
∑PCBs^b^	0.05	−0.05 to 0.15	0.06	−0.05 to 0.16
TEQ	−0.01	−0.10 to 0.08	0.00	−0.10 to 0.09
Inducers^c^	0.09	−0.01 to 0.19	0.11	0.01 to 0.21
Mono-*ortho*^d^	0.01	−0.08 to 0.10	0.01	−0.08 to 0.10
Di-*ortho*^e^	0.08	−0.03 to 0.19	0.09	−0.02 to 0.20
Wolff method				
Group 1^f^	0.05	−0.04 to 0.14	0.06	−0.03 to 0.14
Group 2^g^	0.04	−0.07 to 0.14	0.05	−0.06 to 0.16
Group 3^h^	0.09	0.00 to 0.18	0.11	0.02 to 0.20
Individual congeners				
PCB-18	0.03	−0.05 to 0.10	0.03	−0.04 to 0.10
PCB-28	0.01	−0.06 to 0.08	0.02	−0.06 to 0.08
PCB-44	0.03	−0.05 to 0.10	0.03	−0.04 to 0.11
PCB-49	0.02	−0.06 to 0.09	0.02	−0.05 to 0.09
PCB-52	0.03	−0.05 to 0.11	0.03	−0.05 to 0.11
PCB-66	0.00	−0.08 to 0.08	0.01	−0.07 to 0.09
PCB-74	0.03	−0.07 to 0.12	0.03	−0.06 to 0.13
PCB-99	0.09	−0.00 to 0.18	0.11	0.02 to 0.21
PCB-101	0.08	0.02 to 0.15	0.09	0.03 to 0.16
PCB-118	0.01	−0.08 to 0.11	0.03	−0.07 to 0.13
PCB-138	0.07	−0.01 to 0.15	0.09	0.01 to 0.18
PCB-146	0.06	−0.02 to 0.14	0.07	−0.01 to 0.15
PCB-153	0.07	−0.01 to 0.15	0.08	0.00 to 0.17
PCB-156	0.04	−0.03 to 0.11	0.05	−0.03 to 0.12
PCB-180	0.08	0.00 to 0.15	0.09	0.01 to 0.17
PCB-183	0.12	0.05 to 0.19	0.13	0.05 to 0.20
PCB-187	0.09	0.02 to 0.16	0.09	0.02 to 0.17
PCB-194	0.11	0.03 to 0.19	0.12	0.04 to 0.20
PCB-199	0.14	0.07 to 0.22	0.14	0.07 to 0.22

a Models adjusted for neonatal age at time of heel stick for TSH measurement, gestational age at birth, infant birth-weight, sex and mother’s prepregancy BMI.

b Sum of all PCBs with a detection frequency ≥ 75%.

c Enzyme inducers include PCBs 52, 99, 101, 118, 153, 156, 180, 183, 187, 194, and 199.

d Mono-*ortho* PCBs include PCBs 28, 66, 74, 118, and 156.

e Di-*ortho* PCBs include PCBs 18, 44, 49, 52, 99, 101, 138, 146, 153, 180, and 194.

f Group 1 includes PCBs 44, 49, 52, 101, 187, and 199.

g Group 2 includes PCBs 66, 74, 118, 138, and 156.

h Group 3 includes PCBs 99, 153, 180, and 183.
